# The effectiveness of pressure therapy (15–25 mmHg) for hypertrophic burn scars: A systematic review and meta-analysis

**DOI:** 10.1038/srep40185

**Published:** 2017-01-05

**Authors:** Jin-Wei Ai, Jiang-tao Liu, Sheng-Duo Pei, Yu Liu, De-Sheng Li, Hong-ming Lin, Bin Pei

**Affiliations:** 1 Evidence-Based Medicine Center, Xiangyang Hospital, Hubei University of Medicine, Xiangyang 441000, P.R. China; 2 Department of Plastic Surgery, Xiangyang Hospital, Hubei University of Medicine, Xiangyang 441000, P.R. China; 3 School of Life Sciences, Central China Normal University, Wuhan 430079, P.R. China

## Abstract

Although pressure therapy (PT) represents the standard care for prevention and treatment of hypertrophic scar (HS) from burns, its practice is largely based on empirical evidence and its effectiveness remains controversial. To clarify the effect of PT (15–25 mmHg) for HS, we performed the systematic review and meta-analysis. Several electronic databases were screened to identify related randomized controlled trials (RCTs). 12 RCTs involving 710 patients with 761 HS resulting from burn injuries were included. Compared with non/low-PT, cases treated with PT (15–25 mmHg) showed significant differences in Vancouver Scar Scale score (MD = −0.58, 95% CI = −0.78–−0.37), thickness (SMD = −0.25, 95% CI = −0.40–−0.11), brightness (MD = 2.00, 95% CI = 0.59–3.42), redness (MD = −0.79, 95% CI = −1.52–−0.07), pigmentation (MD = −0.16, 95% CI = −0.32–−0.00) and hardness (SMD = −0.65, 95% CI = −1.07–−0.23). However, there was no difference in vascularity (MD = 0.03, 95% CI = −0.43–0.48). Our analysis indicated that patients with HS who were managed with PT (15–25 mmHg) showed significant improvements. Due to limitations, more large and well-designed studies are needed to confirm our findings and the side-effects of the PT may also need to be evaluated.

Postnatal wound healing after dermal injury is an imperfect process, inevitably resulting in scar formation as the skin re-establishes its integrity^1^. Scar tissue is notably different from the surrounding healthy skin with respect to colour, pigmentation, vascularity, thickness and hardness which may lead to psychological complications such as stigmatization and poor self-esteem^2^,^3^. Scar also causes a range of symptoms including pain, pruritus, erythema and dryness. If it is located close to a joint, scar contracture may result in articular stiffness. So, scars may give rise to cosmetic, symptomatic, psychological and functional problems for patients thereby making a seriously impact on their quality of life^4^,^5^,^6^. Hypertrophic scar (HS) is a very common cutaneous complication following dermal wound, especially after severe burns^7^,^8^. Currently, there are a variety of therapeutic options for treatment of HS including pressure therapy (PT), silicone-based products, intralesional corticosteroids, laser therapy, bleomycin, fluorouracil, topical imiquimod and surgical excision^2^,^9^,^10^. However, no ideal or all-purpose method of scar control exists and HS management remains a problematic challenge for both patients and health care providers^5^,^11^.

PT has been the mainstay of HS treatment since the early 1970s^12^,^13^. Although it represents the standard care for prevention and treatment of HS from burns, there is no scientific evidence for its uses and its practice is largely based on empirical evidence^14^. Firstly, the exact optimal pressure required for effective treatment has never been scientifically established^15^,^16^,^17^. Some studies indicated that 15 mmHg pressure was required to achieve a therapeutic effect^3^,^18^. Pressure less than 15 mmHg (low-PT) may not show the desired effect and a pressure of more than 40 mmHg is more likely to cause severe discomfort and may be potentially harmful^15^. The application of 15–25 mmHg pressure is most commonly used in clinic practice^2^,^19^. Secondly, the effectiveness of PT in HS prevention and treatment remains controversial. Some studies showed that PT could promote HS maturation, restrain its formation, improve its appearance, minimize itch and pain^10^,^20^. However, others indicated that PT not only left the ultimate outcome of burn wounds unchanged but also increased the incidence of overheating, pruritus, blistering, wound breakdown and abnormal bone growth^7^,^18^.

In 2009, a standard meta-analysis was conducted including six randomized controlled trials (RCTs) involving 316 patients. The pooling of data from four studies suggested that PT could only mildly improve scar height but did not appear to alter global scar score (Vancouver Scar Scale score, VSS), pigmentation, vascularity, pliability and colour^21^. Based on the inconsistent study conclusions and the results of the meta-analysis, ambiguous recommendations were given by the recent international clinical guidelines^22^,^23^,^24^. Between 2009 and now, some new relevant RCTs were published. Therefore, we performed the updated systematic review and meta-analysis including all eligible RCTs to reappraise the effect of PT (15–25 mmHg) for HS in burns.

## Methods

This meta-analysis was reported according to the PRISMA guidelines^25^.

### Inclusion and exclusion criteria

A study was included if it met the following criteria: (1) Study type: RCT reported by original articles; (2) Patients: those with second degree burns or more or those with HS from burns; (3) Interventions: PT (15–25 mmHg); (4) Comparators: no pressure (non-PT) or low-PT; (5) Outcomes: VSS, thickness, color, pigmentation, hardness and vascularity. We excluded editorials, brief reports and data that had multiple publications.

### Data sources and searches

We searched PubMed, Cochrane Library (Issue 1, 2016), CNKI (China National Knowledge Infrastructure) and Embase from their inception date up to January 25, 2016. The medical subject headings (MeSH) and free-text words were used. In order to identify grey literatures, we searched the *ClinicalTrial.gov* and OpenGrey (*www.opengrey.eu*). We used Google scholar to find additional records, which were not included in those databases. The reference lists from included studies, reviews and guidelines were also hand-searched. The detailed search strategy is shown in Supplementary File S1. In the course of literature search, no language or other restrictions were set.

### Study selection

Eligible studies were selected by two independent investigators. First, the titles and abstracts were screened to identify all potentially eligible trials. Then, full text was reviewed to further confirm the studies, which met the inclusion criteria. Lastly, repetitive studies were excluded. Disagreements were resolved by discussion.

### Data extraction

Two investigators independently extracted the following items from each eligible studies: (1) basic information: Surname of the first author, country of the investigation and year of publication; (2) characteristics of participants in each study group, including sample size, average age, ethnicity, percentage of total body surface area (%TBSA) and burn site; (3) treatment information: type of interventions, pressure dose, wear time, duration of treatment, follow-up period, number of cases that discontinued treatment and those that were lost during follow-up; (4) clinical outcomes. In case of any conflict, a discussion was carried out to achieve consensus.

### Methodological quality assessment

The methodological quality of each included study was evaluated using the Cochrane Risk of Bias tool^26^. The items included random sequence generation (selection bias), allocation concealment (selection bias), blinding of participants and personnel (performance bias), blinding of outcome assessment (detection bias), incomplete outcome data (attrition bias), selective reporting (reporting bias) and other bias. Two authors assessed the quality of eligible studies independently and discrepancies were resolved by discussion.

### Data analysis

Statistical analysis was undertaken using RevMan5.3 software (Cochrane Collaboration, Copenhagen, The Nordic Cochrane Centre). Inverse variance method in random-effects model was applied in the meta-analysis. Heterogeneity was estimated using the *I*^2^ statistic and Q tests, *I*^2^ < 50% and *P* > 0.1 indicated low risk of heterogeneity. For each of the comparisons, effect sizes for studies using the same outcome measures were pooled using weighted mean difference (MD) and where not applicable, standardized mean difference (SMD) was used. 95% confidence interval (CI) was also calculated for each pooled result. The *Z* test was used to assess the statistical significance of the pooled MD/SMD and two-tailed *P* < 0.05 was considered significant. Sensitivity analyses were performed by using fixed-effect model to evaluate the stability of the result. We constructed funnel plot to assess for the possibility of publication bias. The resulting symmetrical funnel plot indicated low risk of publication bias.

## Results

### Study selection

**Figure 1:**
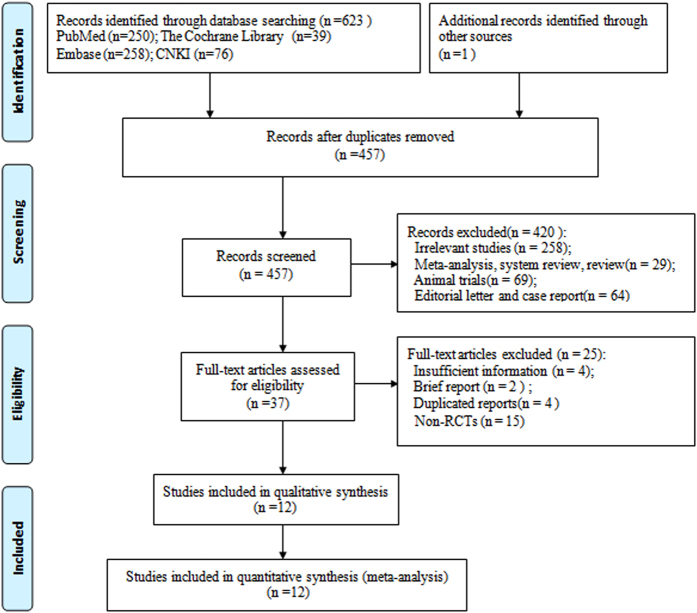
Flow diagram of the selection process for eligible studies.

Our search results yielded 623 records. After excluding duplications, we screened 457 titles and abstracts. A total of 420 records were deleted. This included 258 irrelevant studies, 29 meta-analyses, systematic reviews and reviews, 69 animal studies, 64 editorials and case reports. 37 potentially relevant studies were left for further review of the full-text. We continued to exclude 25 articles: 15 studies for non-RCT study design, 4 studies for insufficient information, 2 brief reports and 4 duplicated reports. 12 studies^27^,^28^,^29^,^30^,^31^,^32^,^33^,^34^,^35^,^36^,^37^,^38^ were ultimately included for meta-analysis. The details of study selection is shown in Fig. 1.

### Study Characteristics and Quality Assessment

**Figure 2:**
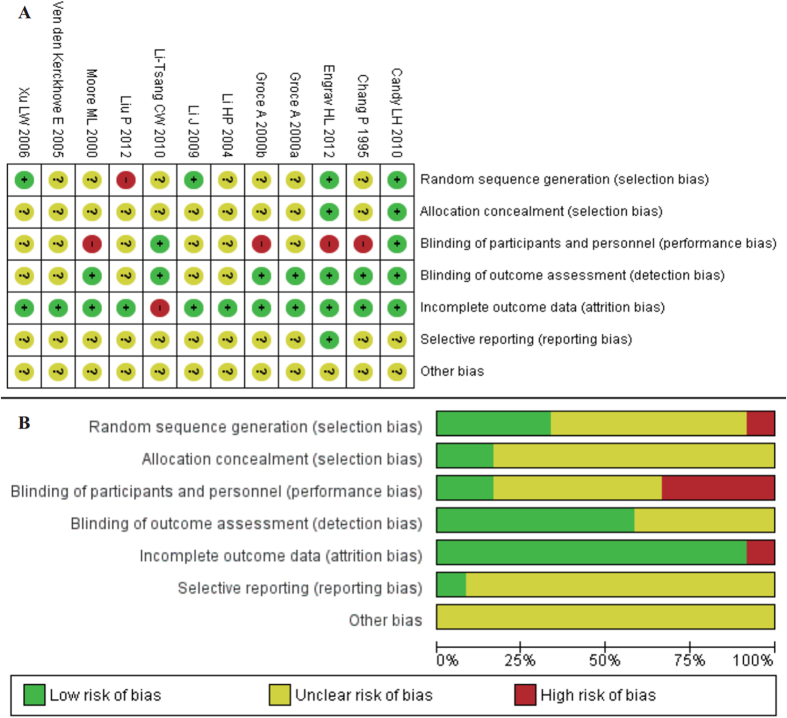
The quality assessment of the included studies. (**A**) Risk of bias summary; (**B**) Risk of bias graph.

The general characteristics of the included studies are reported in Table 1. Eight^27^,^28^,^29^,^30^,^31^,^32^,^33^,^34^ out of the twelve studies were published in English and the others were in Chinese. Twelve studies involved 710 burn patients and the sample-size ranged from 17^29^ to 122^31^. Two studies^27^,^30^ used within-patient design where each patient had at least two wounds, some wounds were/one wound was treated with PT (15–25 mmHg), others wounds/one wound as a control; or in patients with only one wound, one-half of the wound applied PT (15–25 mmHg) and the other-half was considered as control. Ten studies^28^,^29^,^31^,^32^,^33^,^34^,^35^,^36^,^37^,^38^ used between-patient design where each patient was given a treatment. 441, 171, 226 HS were allocated into those treated with PT (15–25 mmHg), low-PT and non-PT groups respectively. Seven of these studies^27^,^31^,^32^,^35^,^36^,^37^,^38^ began PT for burn patients 1 to 3 weeks after wound closure or reepithelialisation. Two studies^28^,^29^ started PT for patients several months post-injury. And three trials^33^,^34^,^36^ did not report the time interval between PT and time of injury. Eleven^27^,^28^,^29^,^31^,^32^,^33^,^34^,^35^,^36^,^37^,^38^ of the twelve trials reported the age of patients, two trials^33^,^34^ included only pediatric patients, five^27^,^29^,^31^,^32^,^37^ studied adults (age ≥18 years) and four^28^,^35^,^36^,^38^ investigated both children and adults. Two studies^34^,^36^ did not mention the burn site while the others were on the limbs. Four studies^27^,^28^,^29^,^36^ reported burn surface area as size dimension, six^31^,^32^,^33^,^34^,^35^,^37^ used %TBSA, while it was not reported in two studies^30^,^38^. Different kinds of pressure garments such as: Medical Z Corporation^®^, Jobst^®^, Urgosyval^®^, Tricolast^®^, *etc* were used in those studies. Four studies^31^,^36^,^37^,^38^ did not clearly describe the manufacturer of pressure garment. Only five studies^27^,^29^,^30^,^32^,^33^ reported the pressure value (scar/garment interface) assessment method. The longest duration of treatment was 12 months^30^ and the shortest 2 months^36^, while one study^31^ did not report the duration of treatment. Endpoint outcomes included VSS score, thickness, color, pigmentation, hardness and vascularity. Three studies^27^,^28^,^35^ had cases lost in the follow-up period and one study^28^ inadequately reported the reasons for losing. The risk of bias figure and the risk of bias summery figure are shown in Fig. 2A,B. Overall, the quality of included studies was under moderate risk of bias.

### Meta-analysis

The results of meta-analysis are displayed in Table 2.

### VSS score

**Figure 3:**
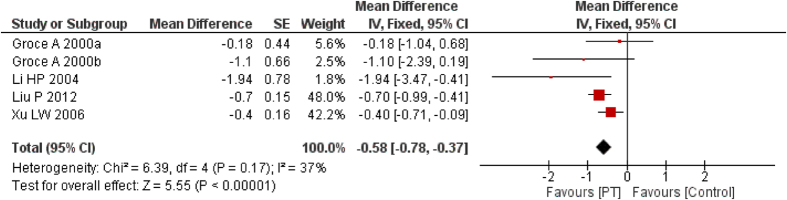
Forest plot of PT vs. non/low-PT in scar VSS score.

VSS score consists of four parameters–pliability, height, vascularity and pigmentation. It is a widely used tool to assess the severity of scar^36^. The total score is 15 and normal skin scores 0. The higher the score, the more severe the scar. Five studies^33^,^34^,^36^,^38^ involving 260 participants reported VSS score. The combined MD showed that PT (15–25 mmHg) could significantly reduce the VSS score (*I*^2^ = 37%, MD = −0.60, 95% CI = −0.92–−0.28, *P* < 0.01, random-effect model). The result varied little when a fixed-effect model was applied (MD = −0.58, 95% CI = −0.78–−0.37, *P* < 0.01) (Fig. 3), which suggested that the result was stable.

### Thickness

**Figure 4:**
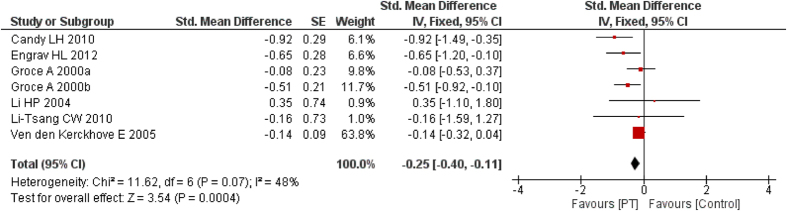
Forest plot of PT vs. non/low-PT in scar thickness.

Eight studies^27^,^28^,^29^,^30^,^32^,^33^,^34^,^36^ compared PT (15–25 mmHg) with non/low-PT to assess the difference in thickness of the scar tissue. One study^30^ that provided no data compared PT (15–25 mmHg) with non-PT and the result was measured using subjective scar assesment (Seattle method) and Ultrasound. However, while Seattle method measured significant difference in scar thickness, ultrasound detected no differences. Of the other seven studies, two^33^,^34^ used VSS and five used ultrasound to assess scar thickness. Pooled result found PT (15–25 mmHg) could significantly reduce the thickness (*I*^2^ = 45%, SMD = −0.38, 95% CI = −0.63–−0.12, *P* < 0.01, random-effect model). The fixed-effect model produced a similar result (*I*^2^ = 45%, SMD = −0.25, 95% CI = −0.40–−0.11, *P* < 0.01) (Fig. 4).

### Colour

**Figure 5:**
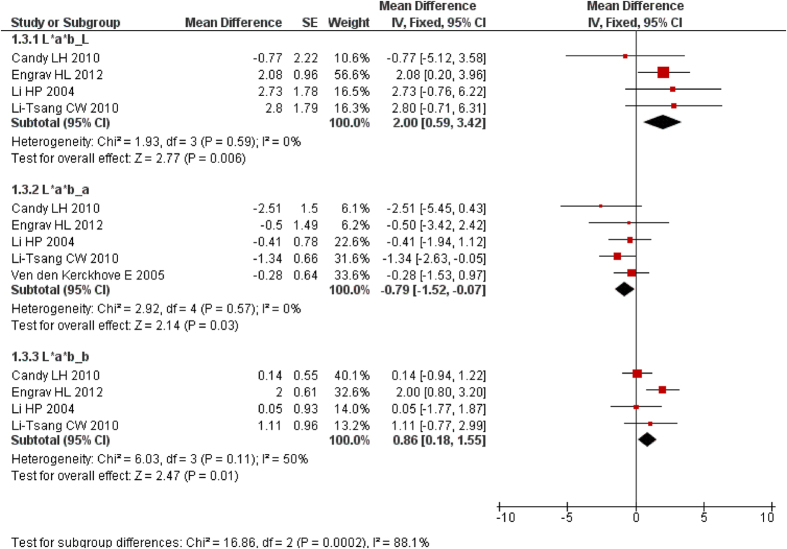
Forest plot of PT vs. non/low-PT in scar colour. L: brightness, (**a**) redness, (**b**) yellowness.

Five studies^27^,^28^,^29^,^32^,^36^ assessed the effect of PT (15–25 mmHg) on scar colour by using L*a*b* color space, in which “L” refers to the brightness, “a” the redness and “b” the yellowness^27^,^29^. Usually, scar tissue is less bright and yellow and redder than normal skin. The results of meta-analysis showed that PT (15–25 mmHg) could improve the cosmetic effect (Fig. 5). L*a*b*: L (*I*^2^ = 0%, MD = 2.00, 95% CI = 0.59–3.42, *P* = 0.01, random and fixed-effect model); a (*I*^2^ = 0%, MD = −0.79, 95% CI = −1.52–−0.07, *P* = 0.03, random and fixed-effect model); and b (*I*^2^ = 50%, MD = 0.86, 95% CI = −0.16–1.87, *P* = 0.10, random-effect model; and MD = 0.86, 95% CI = 0.18–1.55, *P* = 0.01, fixed-effect model). The pooled results were unchanged in sensitivity analysis for increasing brightness and decreasing redness using different effect model thereby indicating that the result is very credible. However, for increasing scar yellowness the result changed significantly showing that the effect of PT (15–25 mmHg) on yellowness needs further investigation.

### Pigmentation

**Figure 6:**
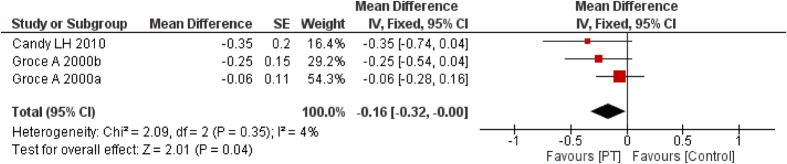
Forest plot of PT vs. non/low-PT in scar pigmentation.

Three studies^29^,^33^,^34^ reported pigmentation based on VSS. For pigmentation, as shown in Fig. 6, *I*^2^ = 4%, MD = −0.17, 95% CI = −0.33–−0.00, *P* = 0.05. The fixed-effect mode showed a similar result, MD = −0.16, 95% CI = −0.32–−0.00, *P* = 0.04.

### Hardness

**Figure 7:**
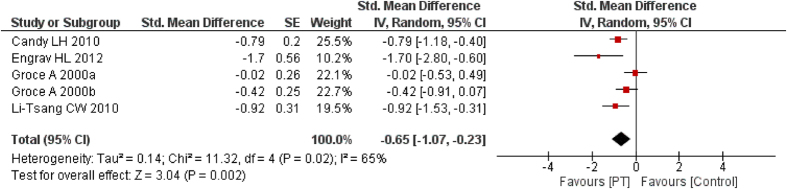
Forest plot of PT vs. non/low-PT in scar hardness.

Six studies^27^,^28^,^29^,^30^,^33^,^34^ observed changes in scar hardness in different treatment groups. Two^33^,^34^ of them measured scar hardness using VSS and the others used durometer. Five^27^,^28^,^29^,^33^,^34^ studies’ data can be pooled and as shown in Fig. 7, PT (15–25 mmHg) decreased the scar hardness (*I*^2^ = 65%, SMD = −0.65, 95% CI = −1.07–−0.23, *P* < 0.01, random-effect model). The result was not significantly changed in fixed-effect model (SMD = −0.60, 95% CI = −0.84–−0. 73, *P* < 0.01). One study^30^ that did not present data indicated that the scars that received PT (15–25 mmHg) were softer than non-PT (P < 0.01) at 9 months post-burn. But the difference disappeared at 12 months.

### Vascularity

**Figure 8:**
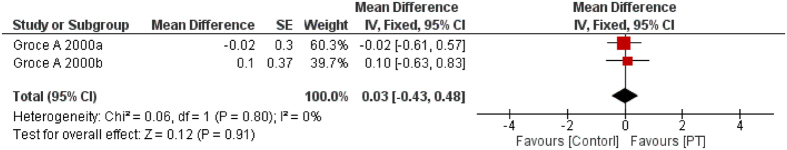
Forest plot of PT vs. non/low-PT in scar vascularity.

Three studies^33^,^34^,^35^ evaluated scar vascularity. The data of one of these studies^35^ which cannot be pooled measured the changes of blood perfusion at the scar tissue by laser Doppler perfusion imaging and found PT (15–25 mmHg) could significantly reduce the blood perfusion of scar tissue compared to low-PT (*P* < 0.05). The other two studies^33^,^34^ assessed the scar vascularity using VSS and the combined result found no significant difference in vascularity (*I*^2^ = 0%, MD = 0.03, 95% CI = −0.43–0.48, *P* = 0.91, random and fixed-effect model) Fig. 8.

### Other outcomes

One of the studies^31^ with 122 burn patients (64 in PT group and 58 in non-PT) that was undertaken to determine the efficacy of PT (15–25 mmHg) showed no significant differences between the two groups when the length of hospital stay and time for wound maturation were compared. Another study^28^ concerned with the scar pain and itch (using Visual Analog Scale, VAS) found that PT had more effectiveness in reducing scar pain than non-PT (*P* = 0.02). However, there was no significant difference in alleviating itching between the two groups^28^.

### Additional analysis

An unpublished trial was included in previous meta-analysis^21^, but we did not obtain the trial in all of our available databases. So, we performed an additional analysis, extracting the trial’s data from the previous study^21^ and recalculating the results. As shown in Supplementary File S2, all related clinical outcomes were unchanged.

### Publication bias

Publication bias was assessed by funnel plot. All funnel plots were generally symmetrical (See Supplementary Fig. S1), indicating low risk of publication bias in our study.

## Discussion

PT represents the standard care for prevention and treatment of HS from burns, but there is no scientific evidence for using it^22^,^23^. Practice is largely based on empirical evidence and its effectiveness remains controversial in current studies^22^. This makes it unfavorable for clinicians to choose effective treatment measures. In this systematic review and meta-analysis, we included all relevant RCTs to compare the therapeutic effects of PT (15–25 mmHg) and non/low-PT. The results indicated that PT (15–25 mmHg) could improve clinical effects including decreasing VSS score, pigmentation, redness and increasing scar brightness. Our results are reliable given that pooled results were unaltered in sensitivity and additional analyses and there was no publication bias in the included studies.

However, for increasing scar yellowness, the result was not stable enough in sensitivity analysis and needs to be investigated further. Similarly, the results of scar thickness, hardness and vascularity may be influenced by the study with no data or inconsistent outcome indicators. One study^30^ that provided no data gave results on scar thickness and hardness in patients who were treated with PT and non-PT for 12 months. The study indicated that PT had positive therapeutical effect at 9 months post-burn, but showed no difference between PT and non-PT at 12 months. The combined result found no significant difference between PT and non/low -PT in vascularity (MD = 0.03, 95% CI = −0.43–0.48, P = 0.91). One study^35^ measured the changes of scar tissue blood perfusion by laser Doppler perfusion imaging and it showed that PT (15–25 mmHg) could significantly reduce the blood perfusion of scar tissue and thus reduce the scar vascularity^35^. However, this study data cannot be pooled.

The overall methodological quality of the included studies was moderate. Eight of the twelve studies did not report the method of randomization and ten had inadequate reports of allocation concealment. There was inadequate blinding in most studies and as previous researches have demonstrated inadequate blinding maybe associated with exaggerated treatment effects^21^. One study^28^ inadequately reported the reasons for withdrawal from treatment. It has been previously shown that the treatment efficacy in patients who withdrew from researches were different from those that completed the trial. Studies that failed to provide details regarding withdrawals were at an increasing risk of producing invalid results^26^. Future investigations should ensure adequate randomization, concealment of allocation, blinding of patients and outcome assessors and descriptions of withdrawals and losing.

To be effective, pressure garment should be worn for at least 23 hours a day and the treatment should be continued for a period of 6–12 months or until the scar matures^9^,^22^,^39^,^40^,^41^. However, the exact optimal pressure required for effective treatment has never been scientifically established^15^. 15–25 mmHg pressure is used mostly in clinical practice. Higher pressures increase the effect and it has also been claimed to give more rapid results in the time of scar maturation^19^. However, pressure above 40 mmHg induces discomfort and potential harm, such as blistering, paresthesia, abnormal bone growth, limb necrosis, etc^12^,^42^. Moreover, higher pressure may also cause higher risk of pressure loss over time and increase the incidence of incompliance^6^,^43^. On the contrary, pressure less than 15 mmHg may appear to have poor/no effect^2^,^28^,^29^. A 12-year long-term high-quality study^27^ demonstrated that 15mmHg was considered to be the minimum effective “dose”. And other previous studies^29^,^32^ suggested that the pressure level required might need to be higher than 15 mmHg. Generally, it is recommended that pressure should be maintained between 15 to 25 mmHg which being above capillary pressure, diminishes the supply of blood and nutrients to the scar tissue and is safer and more effective^2^,^27^. Thus, in this study, we took the non-PT and low-PT as one treatment group to assess the effect of PT (15–25 mmHg) for hypertrophic burn scars.

Although we found that PT (15–25 mmHg) had positive impacts on HS, some confounding factors could influence the therapeutic effects. Firstly, the time to begin PT^39^,^40^,^44^,^45^,^46^. Different time interval between PT and burn-injury may have different PT effects, for example a recently healed burn wound and an established HS may respond differently to PT (15–25 mmHg). Previous studies recommended that PT should be used as soon as the healing skin can tolerate the pressure^39^,^40^,^44^ and/or shear force generated by the intervention^45^,^46^. The earlier the treatment begins the better the outcomes^44^. Secondly, the age of the burn patient^47^,^48^,^49^. Different life stages may have different healing processes and treatment compliances, which may have a great influence on assessing the effect of PT^48^. Thirdly, the location of burn^12^,^50^. Burn in different parts of the body may need different treatment requirements. And pressure is difficult to apply evenly across the body, particularly in concave areas and flexor joints^12^. Fourthly, methods of measuring the outcomes. Inequable validity may be found in different evaluation methods or scales^36^. Last but not least, pressure monitoring. In the course of PT, pressure losing along with therapy could also impact the final treatment effect and thus more attention should be paid on pressure monitoring^29^,^32^. However, in our included studies, the confounding factors were different or unreported and due to insufficient information we could not consider their influence. The effect of PT at different terms post-injury, life stage of patients, parts of the body burned, depth of burn, etc., should be further investigated in future studies. In addition, recognized and/or objective evaluation methods should be utilized and the pressure in scar/garment interface should be monitored in the future investigations.

Several investigations^48^,^51^,^52^ showed that PT had effectiveness in enhancing HS maturation and controlling the itch associated with HS because PT could reduce collagen synthesis and prevent HS formation and contracture. However, others studies^49^,^53^ found PT may cause skin breakdown/ulceration and aggravate the level of itching, especially in summer months. Prolonged wear of pressure garment may affect the skin perspiration and thus create heat and pruritus^49^. Only one of the included studies^31^ focused on length of hospital stay and time of wound maturation for which no significant differences were found between the PT and non-PT group. Another study^28^ found that PT had no more effectiveness in alleviating scar itching than non-PT where VAS score of itching changed from 4.47 ± 2.45 to 2.63 ± 1.91 and from 4.78 ± 3.35 to 3.09 ± 2.34 in PT and non-PT group respectively. Due to limited number of studies the efficacy of PT in scar maturation and itching could not be assessed to the best of its potential.

Some side-effects of PT such as blistering, ulceration, scar breakdown, limb swelling, etc. which are usually caused by too much pressure being applied were reported in previous studies^12^,^54^,^55^. In all of our included studies, 15–25 mmHg pressure, which was considered as safety pressure level was used in PT group. However, other adverse effects such as skeletal deformity, heterotopic ossification, muscle atrophy, joint stiffness^47^,^56^, etc. that could be caused by long-term usage of pressure garment may not have occurred or were unreported in our included studies. Thus, we did not assess the safety of PT. Future studies may need to pay more attention on the adverse-effects of PT. In addition, some studies^12^,^53^ declared that poor appearance of pressure garments made patients feel self-conscious, causing embarrassment and other problems to patients. More RCTs may also need to investigate the impact of PT in patients’ quality of life. In this way, the effects of PT may be investigated more comprehensively and accurately.

The exact mechanisms of how pressure positively influences the scar outcome following burns are not fully understood^7^,^8^,^9^. However, it is widely believed that the pressure can control collagen synthesis, facilitate scar maturation and reduce scar redness by limiting the supply of blood, oxygen and nutrients to the scar tissue^28^,^29^. Pressure has been also postulated to reduce the levels of collagen production more rapidly than the natural maturation process^15^. Mechanical loading induces alteration in collagen fiber turnover, remodeling and realignment and the reduced development of whorled collagen nodules result in the thinning and softening of scar tissues^7^,^12^,^16^. In addition, it is accepted that application of pressure commonly alleviates the itch and pain associated with active hypertrophic scars^42^,^43^.

In 2009, a standard meta-analysis^21^, included six RCTs, involving 316 patients, pooling four studies’ data suggested that PT when compared non/low-PT could only mildly improve scar height (SMD = −0.31, 95% CI = −0.63 to 0.00, *P* = 0.05), but could not to alter global scar scores (VSS), pigmentation, vascularity, pliability and colour. Based on the current inconsistent study conclusions and the results of Meta-analysis, an international clinical recommendation^23^ in 2014 concluded: “The long-time standard care for prevention and treatment of hypertrophic scars from burns is largely based on empirical evidence; no change in global scar scores and only small improvement in scar height was reported in meta-analysis; low pressure is less effective than high-pressure treatments; and patients with moderate or severe scarring experienced greater clinical benefit”^23^. And another useful guide^22^ commented: “PT alleviates itching and pain associated with abnormal scars but with no scientific evidence in the use of pressure garments; given current lack of evidence, well-designed clinical trials are required to examine the effectiveness, risks and costs of PT”^22^.

The sample-size of previous meta-analysis^21^ was relatively small and the conclusions were not stable enough. From 2009 to now, five new relevant RCTs were carried out. Therefore, we reappraised the effect of PT (15–25 mmHg) for HS in burns. The results of our study were significantly different from the previous meta-analysis. The sample-size was larger, the pooled results were unaltered in sensitivity analysis and there was no obvious publication bias in the included studies. More interestingly, we added an unpublished trial data in our study and all related clinical outcomes were found to be unchanged. So, our results are more reliable.

However, it is important to note the limitations of our study. Firstly, our results may be influenced by the small number of included studies, the limited sample-size and inconsistent clinical outcomes of each study. Secondly, due to insufficient data, our study did not consider the %TBSA, burn degree and burn site although the different %TBSA, burn degree and burn site may have varying efficacy. Thirdly, since long-term follow-up studies were rare, our study failed to analyze the prospective efficacy of PT. Fourthly, none of the included studies studied adverse effects and we were unable to assess the safety of PT. Last but not least, our analysis suffered in quality of included studies because most of the studies did not describe the allocation concealment and blinding method, which may exaggerate the treatment effects, especially in subjective outcomes.

## Conclusions

Our meta-analysis demonstrated that burn patients managed with PT (15–25 mmHg) showed significant improvements. Due to the limitations of the current studies, larger and well-designed studies are needed to confirm our findings. Furthermore, the side-effects of PT may also need to be evaluated in the future.

## Additional Information

**How to cite this article**: Ai, J.-W. *et al*. The effectiveness of pressure therapy (15–25 mmHg) for hypertrophic burn scars: A systematic review and meta-analysis. *Sci. Rep.* **7**, 40185; doi: 10.1038/srep40185 (2017).

**Publisher's note:** Springer Nature remains neutral with regard to jurisdictional claims in published maps and institutional affiliations.

## Supplementary Material

Supplementary Information

## Figures and Tables

**Table 1:** Characteristics of the studies included in this meta-analysis.

Study	Study design	Sample size/(PT/Control)	Average age (year)	Burn area or %TBSA	Details in PT group	Time to PT	Type of PT used/Method of ssessment	Control group	Flow-up	With-draw	Outcomes
Engrav 2012	Within wounds	54 (54/54)	36 ± 14	≥4 cm in diameter	25.00 ± 6.3 mmHg 23 h per day	≥3 weeks to heal	Medical Z Corporation^®^/I-Scan system	6.40 ± 6.2 mmHg 23 h per day	9.5	11	Thickness; Hardness; Color
Li-Tsang 2010	Between patien	104 (59/45)	22 ± 19	>16 cm^2^	15~25 mmHg 24 h per day except bathing time	14.9 ± 30.8 months post-injury	Tailor-made pressure garment/Not report	Non-PT	6	20	Thickness; Hardness; Color; Pain; Pruritus
Candy 2010	Between patient	17 (53HS) (25/28)	26 ± 8	>4 cm × 4 cm	23.23 ± 1.11 mmHg 23 h per day except for hygienic measures	5.21 ± 1.91 months post-injury	Tailor-made plastazote paddings/Pliance X System	14.53 ± 1.0 mmHg 23 h per day except for hygienic measures	5	0	Thickness; Hardness; Color
Moore 2000	Within wounds	23 (23/23)	Not report	Not report	Mean = 25 mmHg 23 h per day except bathing time	healed burn wound	Medical Z Corporation^®^/I-Scan system	Non-PT	12	0	Thickness; Hardness; Color
Chang 1995	Between patient	122 (64/58)	31 ± 2	(21.7 ± 2.2)%	15~25 mmHg	healed burn wound	Pressure garment/Not report	Non-PT	Not report	0	Length of stay; Wound maturation time
Ven den Kerckhove 2005	Between patient	60 (75HS) (41/34)	37.5 (19~6)	8.5% (1~30%)	19.75 ± 3.44 mmHg 23 h per day	2 weeks after reepithelialisation	Tricolast^®^ or Anvarex^®^/ENV 12718	11.85 ± 2.41 mmHg 23 h per day	3	0	Thickness; Color
Groce 2000a	Between patient	50 (25/25)	6.6 (1~7)	48.3% (11~99%)	24.7 ± 8.5 mmHg	Not report	Jobst^®^/Tek-Scan Matscan	10.4 ± 7.6 mmHg	6	0	VSS; Thickness; Hardness; Pigmentation; Vascularity
Groce 2000b	Between patient	28 (10/18)	8.2 (1~6)	11.2% (1~30%)	Mean = 21.8 mmHg	Not report	Jobst^®^/Not report	Non-PT	6	0	VSS; Thickness; Hardness; Pigmentation; Vascularity
Li 2009	Between patient	60 (30/30)	37 (14~52)	10~50%	10~25 mmHg 23.5 h per day	1 week after wound closure	Urgosyval^®^/Not report	5~10 mmHg 23.5 h per day	6	2	Scar tissue perfusion
Li 2004	Between patient	43 (34/9)	21 ± 19	(7.13 ± 4.77) cm × (4.14 ± 2.94) cm	24~25 mmHg 24 h per day except bathing time	Not report	Pressure garment/Not report	Non-PT	2	0	VSS; Thickness; Hardness; Color
Zhu 2012	Between patient	62 (31/31)	34 ± 13	10~50%	15~25 mmHg 24 h per day except bathing time	2~ week after wound closure	Pressure garment/Not report	Non-PT	6	0	VSS
Xu 2006	Between patient	87 (45/42)	5~50	Not report	15~25 mmHg 24 h per day except bathing time	2~3 week after wound closure	Pressure garment/Not report	Non-PT	6	0	VSS

HS: Hypertrophic scar; PT: Pressure therapy; Non-PT: Pressure therapy not using; %TBSA: percentage of total body surface area; VSS: Vancouver Scar Scale score.

**Table 2:** A summary results of the meta-analysis.

Outcomes	NO. of study	Heterogeneity test		Effect Model	Effect size		
		*I*^2^	*P*		MD/SMD	95% CI	*P*
VSS	5	37%	0.17	Random	−0.60	−0.92, −0.28	<0.01
				Fixed	−0.58	−0.78, −0.37	<0.01
Thickness	7	48%	0.09	Random	−0.38	−0.63, −0.12	<0.01
				Fixed	−0.25	−0.40, −0.11	<0.01
L*a*b*: L	4	0%	0.59	Random	2.00	0.59, 3.42	0.01
				Fixed	2.00	0.59, 3.42	0.01
L*a*b*: a	5	0%	0.57	Random	−0.79	−1.52, −0.07	0.03
				Fixed	−0.79	−1.52, −0.07	0.03
L*a*b*: b	4	50%	0.11	Random	0.86	−0.16, 1.87	0.10
				Fixed	0.86	0.18, 1.55	0.01
Pigmentation	3	4%	0.35	Random	−0.17	−0.33, −0.00	0.05
				Fixed	−0.16	−0.32, −0.00	0.04
Hardness	5	65%	0.02	Random	−0.65	−1.07, −0.23	<0.01
				Fixed	−0.60	−0.84, −0.37	<0.01
Vascularity	2	0%	0.80	Random	0.03	−0.43, 0.48	0.91
				Fixed	0.03	−0.43, 0.48	0.91

VSS: Vancouver Scar Scale score; L*a*b*: L: Scar brightness; L*a*b*: a: Scar redness; L*a*b*: b: Scar yellowness; MD: mean difference; SMD: standardized mean difference.
