# Supplementary material for: The effectiveness of pressure therapy (15–25 mmHg) for hypertrophic burn scars: A systematic review and meta-analysis
Source: Sci Rep. 2017 Jan 5;7:40185. doi: 10.1038/srep40185 (PMC5215680; doi:10.1038/srep40185)
Supplement: Supplementary Information [file srep40185-s1.doc]

**The effectiveness of** **pressure therapy (15-25 mmHg) for hypertrophic burn scars: A** **systematic review and meta-analysis**

Jin-Wei Ai, Jiang-tao Liu, Sheng-Duo Pei, Yu Liu, De-Sheng LI, Hong-ming Lin , Bin Pei

**Supplementary File S1.**

***The detailed search strategy in PubMed***

#1 "Cicatrix, Hypertrophic"[Mesh]

#2 "Hypertrophic Cicatrix"

#3 "Hypertrophic Cicatrices"

#4 "Hypertrophic Scar"

#5 "Hypertrophic Scars"

#6 "Burn Scar"

#7 "Burn Scars"

#8 Keloid

#9 "Thermal injury"

#10 Burns

#11 Burn

#12 #1 OR #2 OR #3 OR #4 OR #5 OR #6 OR #7 OR #8 OR #9 OR #10

#13 "Pressure Therapy"

#14 "Pressure Therapies"

#15 "Pressure Garment Therapy"

#16 "Pressure Garment Therapies"

#17 "Pressure Garment"

#18 "Pressure Garments"

#19 #13 OR #14 OR #15 OR #16 OR #17 OR #18

#20 #12 AND #19 (Outcome: **250**)

***The complete search strategy in PubMed***: (((("Cicatrix, Hypertrophic"[Mesh]) OR ((((((((("Hypertrophic Cicatrix") OR "Hypertrophic Cicatrices") OR "Hypertrophic Scar") OR "Hypertrophic Scars") OR "Burn Scar") OR "Burn Scars") OR Keloid) OR Burns) OR Burn)) OR "Thermal injury")) AND (((((("Pressure Therapy") OR "Pressure Therapies") OR "Pressure Garment Therapy") OR "Pressure Garment Therapies") OR "Pressure Garment") OR "Pressure Garments")

***The detailed search strategy in Cochrane Library***

#1 [Cicatrix, Hypertrophic] explode all trees

#2 "Hypertrophic Cicatrix" or "Hypertrophic Cicatrices" or "Hypertrophic Scar" or "Hypertrophic Scars" or "Burn Scar" (Word variations have been searched)

#3 "Burn Scars" or Keloid or "Thermal injury" or Burns or Burn (Word variations have been searched)

#4 #1 or #2 or #3

#5 "Pressure Therapy" or "Pressure Therapies" or "Pressure Garment Therapy" or "Pressure Garment Therapies" or "Pressure Garment" (Word variations have been searched)

#6 "Pressure Garments" (Word variations have been searched)

#7 #5 or #6

#8 #4 and #7 (Outcome: **39**)

***The detailed search strategy in Embase***

#1 'hypertrophic scar'/exp

#2 'hypertrophic scars'

#3 'hypertrophic cicatrix'

#4 'hypertrophic cicatrices'

#5 'burn scar'/exp OR 'burn scar'

#6 'burn scars'

#7 'keloid'/exp OR keloid

#8 'thermal injury'/exp OR 'thermal injury'

#9 'burns'/exp OR burns

#10 'burn'/exp OR burn

#11 #1 OR #2 OR #3 OR #4 OR #5 OR #6 OR #7 OR #8 OR #9 OR #10

#12 'pressure therapy'

#13 'pressure therapies'

#14 'pressure garment therapy'

#15 'pressure garment therapies'

#16 'pressure garment'

#17 #12 OR #13 OR #14 OR #15 OR #16

#18 #11 AND #17 (Outcome: **258**)

**Supplementary File S2.**

Additional analysis†

| Outcomes | NO. of study | Heterogeneity test | | Effect  Model | Effect size | | |
| --- | --- | --- | --- | --- | --- | --- | --- |
|  |  | *I*^2^ | *P* |  | MD/SMD | 95% CI | *P* |
| VSS | 6 | 23% | 0.26 | Random | -0.58 | -0.85, -0.31 | < 0.01 |
|  |  |  |  | Fixed | -0.57 | -0.77, -0.37 | < 0.01 |
| Thickness | 8 | 41% | 0.11 | Random | -0.34 | -0.58, -0.10 | 0.01 |
|  |  |  |  | Fixed | -0.25 | -0.39, -0.11 | < 0.01 |
| Pigmentation | 4 | 40% | 0.17 | Random | -0.22 | -0.43, -0.00 | 0.05 |
|  |  |  |  | Fixed | -0.19 | -0.34, -0.03 | 0.02 |
| Hardness | 6 | 61% | 0.02 | Random | -0.57 | -0.95, -0.20 | < 0.01 |
|  |  |  |  | Fixed | -0.55 | -0.78, -0.33 | < 0.01 |
| Vascularity | 3 | 0% | 0.75 | Random | -0.10 | -0.39, 0.19 | 0.48 |
|  |  |  |  | Fixed | -0.10 | -0.39, 0.19 | 0.48 |

^†^, Included the unpublished trial data in this meta-analysis; MD: mean difference; SMD: standardized mean difference

**Supplementary Figure. S1.**

***Funnel plots for assessment the possibility of publication bias*.**


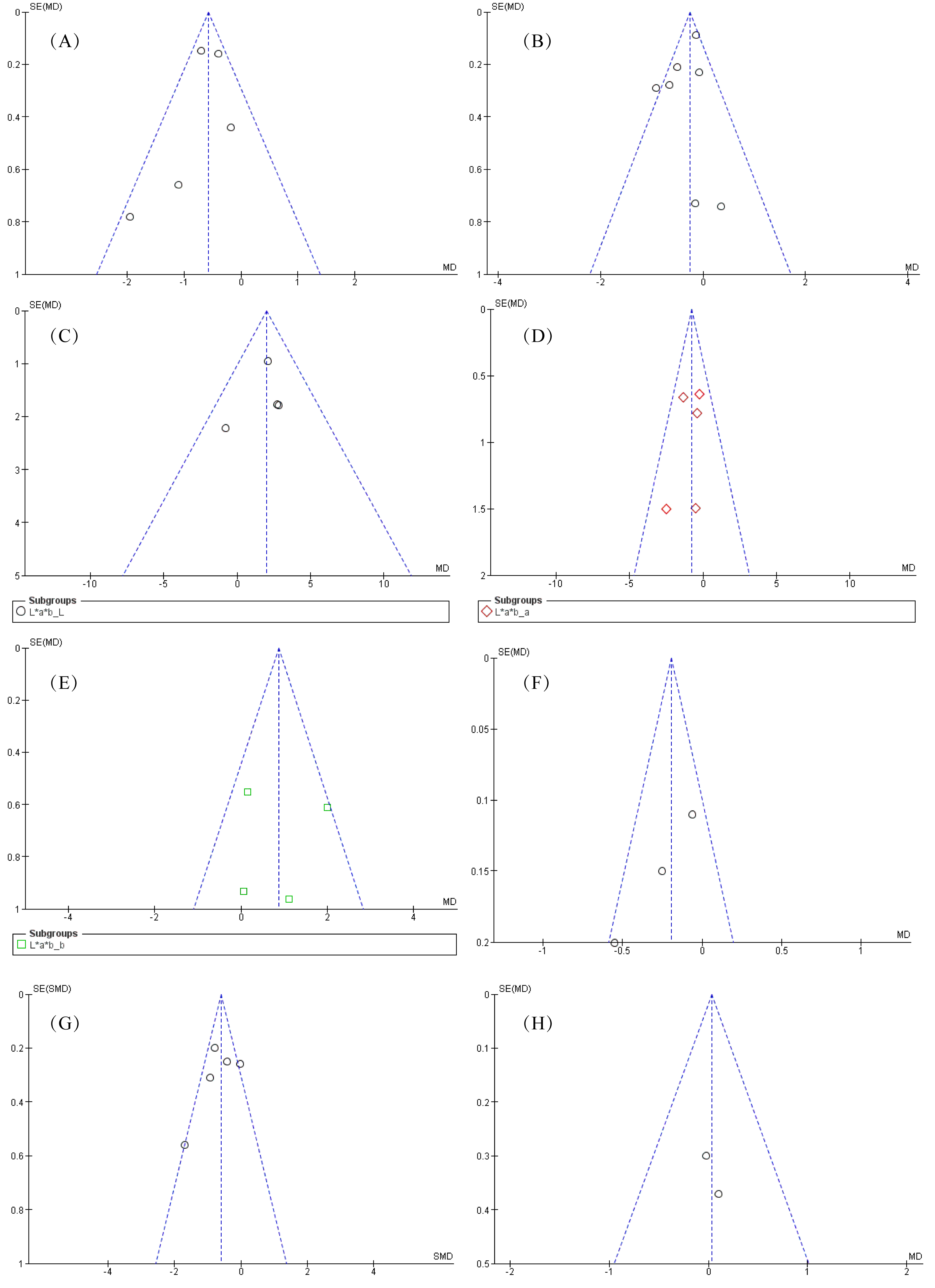


Funnel plots of: (A) Vancouver Scar Scale score; (B) Scar thickness; (C) Scar brightness; (D) Scar redness; (E): Scar yellowness; (F) Scar pigmentation; (G) Scar hardness; (H) Scar vascularity
